# *AtHKT1* gene regulating K^+^ state in whole plant improves salt tolerance in transgenic tobacco plants

**DOI:** 10.1038/s41598-018-34660-9

**Published:** 2018-11-08

**Authors:** Li Wang, Yuhui Liu, Shoujiang Feng, Zhuoyu Wang, Jinwen Zhang, Junlian Zhang, Di Wang, Yantai Gan

**Affiliations:** 1 Gansu Provincial Key Laboratory of Aridland Crop Science, Gansu Key Laboratory of Crop Genetic and Germplasm Enhancement, Gansu Agricultural University, Lanzhou, 730070 China; 2 College of Life Science and Technology, Gansu Agricultural University, Lanzhou, 730070 China; 3 College of Horticulture, Gansu Agricultural University, Lanzhou, 730070 China; 4 Institute of Soil, Fertilizer and Water-saving Agriculture, Gansu Academy of Agricultural Sciences, Lanzhou, 730070 China; 5 College of Agronomy, Gansu Agricultural University, Lanzhou, 730070 China; 6 Centre de Recherche CHUM, Montreal, H2X0A9 Canada; 7 Swift Current Research and Development Centre, Agriculture and Agri-Food Canada, Swift Current, S9H3X2 Canada

## Abstract

The status of K^+^ is important for plant health. However, little is known about if high-affinity potassium transporter HKTs may help K^+^ retention under salt stress. Here, we determined the effect of *Arabidopsis thaliana* transporter gene (*AtHKT1*) on the K^+^ status, Na^+^-induced toxicity, and salt tolerance in tobacco (*Nicotiana tabacum* L.). Six *AtHKT1* transformed tobacco lines (T1, T2, … T6) were contrasted with a non-transgenic plantlet at the whole-plant and molecule levels. *AtHKT1* gene was expressed in the xylems of stem, root and leaf vein in the transgenic tobacco, with the line T3 having highest expression. At Day 15, in the 200 mmol L^−1^ NaCl stress treatment, the transgenic plants remained a healthy K^+^ status, while the control plants decreased K^+^ content by 70% and Na^+^ contents in leaves and stems were 1.7 times that in the transgenic line. The *AtHKT1* expression enhanced the activities of SOD, CAT and POD, raised chlorophyll and soluble sugar contents and root activity, and decreased MDA and proline contents and electrolyte leakage destruction. The constitutive over-expression of *AtHKT1* that helps maintain a healthy K^+^ status while reducing Na^+^ toxicity may serve as a possible mechanism in maximizing productivity of tobacco under salt stress.

## Introduction

Soil salinization is a severe trouble that threatens agricultural development worldwide^1,2^. High salt contents in soils can stunt plant growth due to osmotic stress^3^, ionic imbalance or ionic toxicity^4,5^. Regulation of K^+^ in plants has been reported to be the most important mechanism for maintaining of physiological concentrations of cytoplasm and improving salinity tolerance^6^. The challenge is that it is difficult to increase K^+^ uptake under salt-stress conditions in most crops. Some halophytes have a strong competitive edge in keeping K^+^ steady state under high Na^+^ stress^7^, suggesting the importance of high affinity potassium transporter (HKT) in K^+^ transport under Na^+^ stress^7–9^.

*HKTs* genes are found in microorganisms and plants^10^, and which is classified of the Trk/Ktr/HKT transporter superfamily. HKTs are activated in plasma membrane^11^, and make an major duty in the K^+^ and Na^+^ transportation in higher plant^11–16^. In halophytes, *Puccinellia tenuiflora* PutHKT2;1 is able to uptake K^+^ in the media with high NaCl or low K^+^ concentrations^7^. *Thellungiella salsuginea* TsHKT1;2, has forceful selectivity with the ability to transport K^+^ over Na^+^ ^8^. In crops, *Oryza sativa* OsHKT1;5 is involved in taking back Na^+^ from transpiration stream, preventing further transport of Na^+^ to the leaves^17^. *Triticum aestivum* TmHKT1;5-A can withdraw Na^+^ from the xylem and decrease transportation of Na^+^ to leaves^18^. Transgenic tobacco (*Nicotiana tabacum* L.) plants are reported to increase root length and fresh weight and enhance salt tolerance through over-expression of *Zea mays ZmHKT1;1a* or *ZmHKT1;1b*^19^. However, some reverse results are also reported in other higher plants. For instance, the *TaHKT2* gene down-regulation in salt could confer wheat resistance to salt^20^. Knocking out the *HKT1* gene enables to maintain the level of salinity resistance in wheat^21^.

The crucifer model plant *Arabidopsis thaliana* has only one HKT homologous protein AtHKT1^22^. Most studies support the model that AtHKT1 regulates Na^+^ accumulation in stem, as AtHKT1, localized in the plasma membrane of xylem parenchyma cells, unloads Na^+^ from the xylem to the xylem parenchyma cells in both stems and roots, thus decreasing Na^+^ transport from root to stem and reducing Na^+^ movement into leaf cells^23–28^. While over-expressing in the cortex and epidermis, the native *AtHKT1* gene is up-regulated in the transgenic *Arabidopsis thaliana* plants^29^. In these studies, the change of *AtHKT1* gene expression and Na^+^ accumulation are often deemed to be the main responses to salt stress. *Arabidopsis thaliana* and *Thellungiella salsuginea* are in the same family, and AtHKT1may share some similar functions as TsHKT1;2 in K^+^ transport^8^. K^+^ status was thought as a vital reason of plant survival to salt. However, little has been reported if *AtHKT1* gene might be involved in the K^+^ accumulation in heterologous plants^30^. An unanswered question is that: could *AtHKT1* gene directly improve K^+^ accumulation in heterologous plants under high Na^+^ stress conditions and thus improving plant tolerance to the stress?

In the present study, a high-affinity potassium transporter (HKT) gene - *AtHKT1* was identified in *Arabidopsis thaliana*, and *AtHKT1:GFP* fusion genes were transferred to a tobacco cultivar – ‘CV87’ driven by the CaMV 35S promoter. We hypothesize that (i) the *AtHKT1*-transformed tobacco plantlets have an improved resistance to salt contrast with non-transgenic tobacco, and (ii) the over-expression of *AtHKT1* gene supports keeping plant K^+^ accumulation under salt stress. To examine the hypotheses, we made a series of investigations, involving (i) cloning *AtHKT1* gene, constructing *AtHKT1:GFP* fusion genes vector and obtaining transgenic tobacco plants; (ii) testing and verifying *AtHKT1:GFP* expression; (iii) determining the whole-plant K^+^ and Na^+^ contents; and (iv) assessing biophysicochemical traits, such as the contents of total chlorophyll, soluble sugar, and proline, as well as MDA, electrolyte leakage, antioxidant enzyme activity, root activity and tissue biomass. These estimations hold out the hypothesis that the continuous over-expression of *AtHKT1* gene assists keeping plantlet K^+^ steady state, alleviates the Na^+^ ion toxic, and improves plant tolerance to salt stress.

## Results

### Clone and bioinformatics of *AtHKT1* gene in *Arabidopsis thaliana*

**Figure 1:**
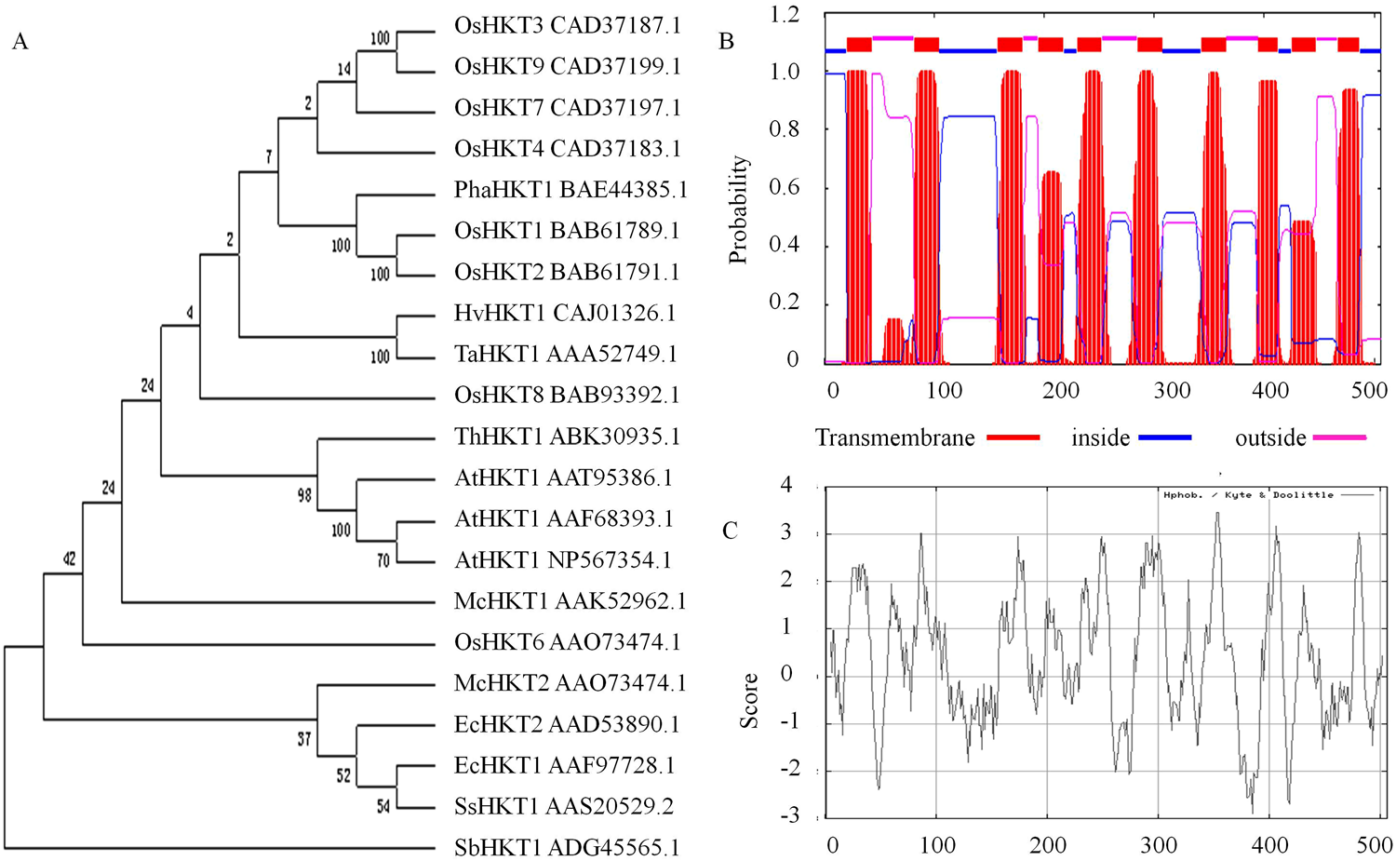
Bioinformatics analysis. (**A**) Phylogenetic tree analysis of AtHKT1 protein form *Arabidopsis thaliana* with other species *HKT* proteins Os: *Oryza sativa*; Pha: *Phragmites australi*; Hv: *Hordeum vulgare*; Ta: *Triticum aestivum*; Th: *Thellungiella halophil*; At: *Arabidopsis thaliana*; Mc: *Mesembryanthemum crystallinum*; Ec: *Eucalyptus camaldulensis*; Ss: *Suaeda salsa*; Sb: *Salicornia bigelovii*. (**B**) Analysis of transmembrane region. (**C**) Analysis of hydrophobicity of AtHKT1 polypeptide.

The cDNA sequence of the *AtHKT1* gene was amplified in *Arabidopsis thaliana*. The coding region was 1521 bp, which encoded a 506 amino acid polypeptide. Sequence alignment analysis showed that the *AtHKT1* gene sequence was 99.3% homologous to the sequence of the *Arabidopsis thaliana AtHKT1* gene (accession No. AF237672) in NCBI. The similarity percentage was 84.0% with *Thellungiella halophil* HKT1 gene sequence (EF025500). The phylogenetic tree shows that AtHKT1 and *Thellungiella halophil* HKT1 (ABK30935.1) are in the same cluster (Fig. 1A). The AtHKT1 is a stable alkaline protein with a possible theoretical isoelectric point of 9.33. One TrkH cation transporter protein conserved domain is at the 152–500 amino acids. The AtHKT1 also has ten transmembrane regions (Fig. 1B), five hydrophilic regions and thirteen hydrophobic regions with uniform distribution in the peptide (Fig. 1C). Thereby, *AtHKT1* gene encoded protein is a transmembrane hydrophobic protein with the characteristics of vector transport protein.

### Vector construction and tobacco genetic transformation

**Figure 2:**
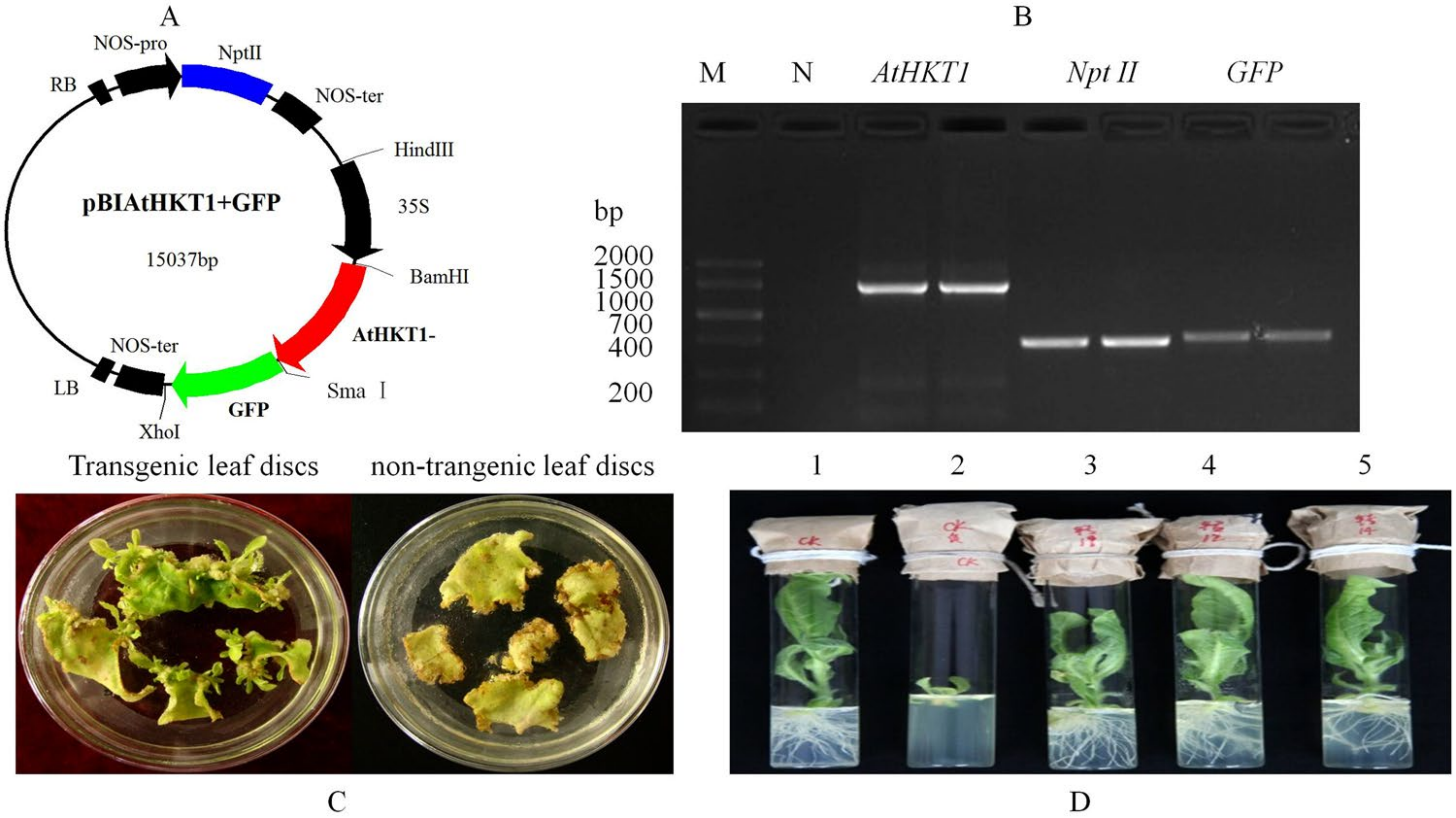
Vector construction and genetic transformation. (**A**) Schematic diagram of the expression vector pAtHKT1 + GFP. *Npt II*, neomycin phosphotransferase II gene; *35S*, cauliflower mosaic virus 35S promoter; *AtHKT1*, *Arabidopsis thaliana* high affinity K^+^-transporter gene; *GFP*, green fluorescent protein gene; *Hin*d III, *Bam* H I, *Sma* I, and *Xho* I are restriction endonuclease recognition sites. (**B**) Verification of the plasmid pAtHKT1 + GFP by PCR amplification of *AtHKT1* gene, *Npt II* gene and *GFP* gene. M, DNA Marker V; N, PCR products of ddH_2_O. (**C**) Formation of shoots directly from leaf discs of the tobacco cultivar CV87 after 4 weeks of growing in the selective medium (MS medium containing 0.50 mg L^−1^ 6-BA + 0.05 mg L^−1^ NAA) supplemented with 100.00 mg L^−1^ kanamycin and 250.00 mg L^−1^ carbenicillin, and (**D**) formation of roots about 7 days in the selective rooting medium (MS medium containing 100.00 mg L^−1^ kanamycin and 200.0 mg L^−1^cephalosporin). 1, non-transgenic tobacco plant in MS medium; 2, non-transgenic control plant in selective rooting medium; 3 to 5, transgenic lines in rooting selective medium. The grouping of gels cropped from same part of the same gel.

The plasmid vector pAtHKT1 + GFP including the *AtHKT1*, *Npt II* and *GFP* genes was constructed and confirmed by PCR amplification (Fig. 2B). Green shoots were grown from the transformed leaf discs after four to five weeks growing in the selective medium (Fig. 2C). After seven days the shoots were shifted to the rooting selective medium, roots were formed (Fig. 2D). But these traits were not found in the non-transgenic plant. Tobacco plants with healthy-grown roots were reproduced for next molecular test.

### Molecular test and gene expression analysis

**Figure 3:**
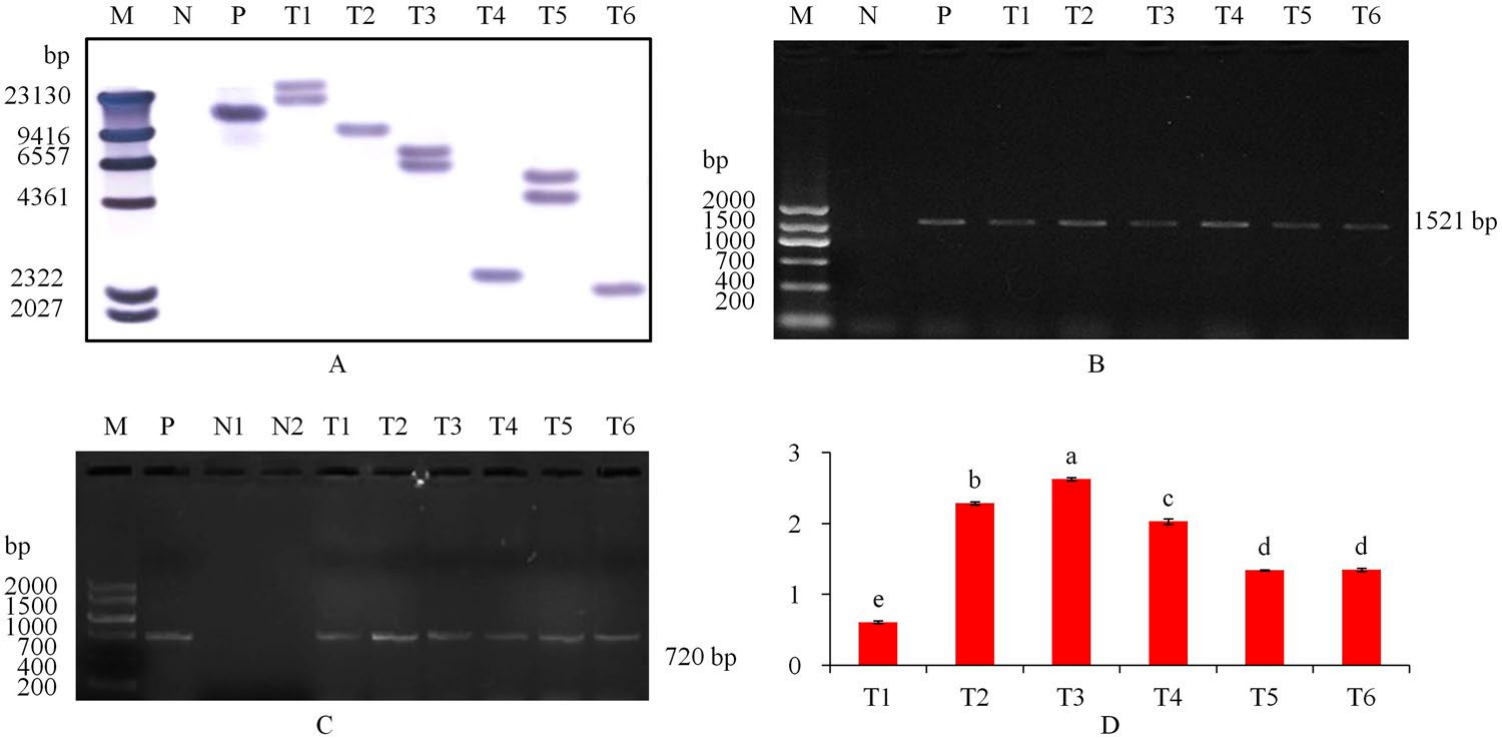
Molecular detection. (**A**) Southern blot analysis of genomic DNA from PCR-positive transformed tobacco lines. The genomic DNA was digested with *Hin*d III and hybridized with a *AtHKT1* probe labeled with digoxigenin. (**B**) verification of transgenic plants by RT-PCR of *AtHKT1* gene. (**C**) RT-PCR of *GFP* gene. (**D**) expression of *AtHKT1* gene with qRT-PCR in transgenic (T1 to T6) tobacco plants. Tukey’s HSD test at *P* < 0.05 was used in analysis of significant differences among means of different transgenic potato plants. The standard errors are shown in line bars (n = 9, i.e., 3 plants × 3 replications). M, DIG DNA Marker II in A and DNA Marker V in B and C; N1, untransformed tomato plant as a negative control; N2, ddH_2_O as a negative control; P, plasmid AtHKT1-GFP as a positive control; T1 to T6, transgenic tobacco lines. The grouping of gels/blots cropped from same part of the same gel.

Applying *AtHKT1* gene-specific primers, PCR analysis revealed that the transformed plants obtained a 1521 bp amplification product and the control plant missed it (Supplementary Fig. S1A). PCR - Southern blot analysis further confirmed the PCR amplification results, which revealed that nine transformed plantlets had the 1521 bp *AtHKT1* gene produc, and the control plants did not have (Supplementary Fig. S1B). The Southern blot further showed that *AtHKT1* gene was confirmed into the six transgenic tobacco line genomes with hybridization signals, and three of the transgenic lines had two copies of the *AtHKT1* gene (Fig. 3A). The mRNA expression in the plant leaves showed that the *AtHKT1* and *GFP* genes were expressed in the six transgenic plants and no transcript was observed in the non-transgenic plants (Fig. 3A,B). The qRT-PCR analysis revealed that all the transgenic lines expressed *AtHKT1* gene. The T3 line had the greatest *AtHKT1* expression, which was up to 4.4 times that of the five other transgenic plant lines (Fig. 3D).

**Figure 4:**
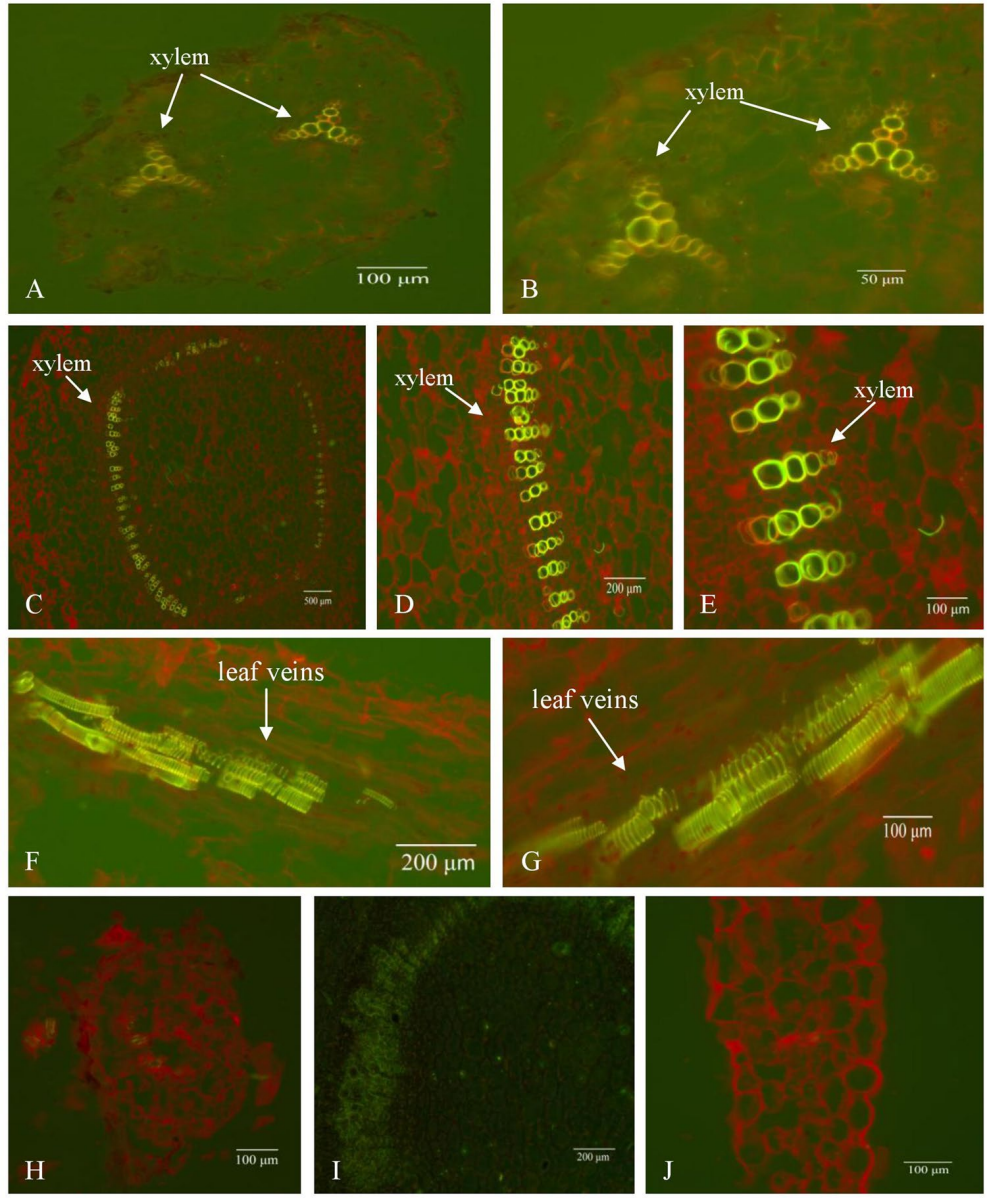
*GFP* gene expression in transgenic tobacco (T3). (**A,B**) root, (**C**–**E**) stem, (**F,G**) leaf. (**A-G**) transgenic tobacco (T3), (**H**–**J**) non-transgenic control (CK). 5 × magnification in (**C**), 10 × magnification in (**A,D,F,H–J**) and 20 × magnification in (**B,E,G**).

To further understand the expression site of the foreign *AtHKT1*:*GFP* gene in the transgenic tobacco, the paraffin sections of the transgenic tobacco were observed under the excitation of blue light. The green fluorescence mainly lied in the xylem of the root, stem and leaf veins in the transgenic lines (Fig. 4A–G). These observations suggest that the fusion gene has undergone through normal transcription and translation and may be involved in the transport of moisture and inorganic salt ions in the conducting tissues.

### Biomass in transgenic tobacco carrying *AtHKT1* gene

**Figure 5:**
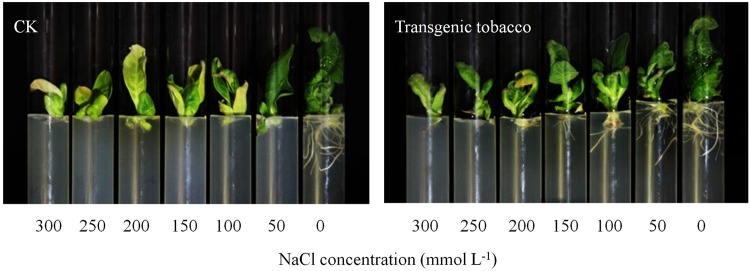
Growth characteristics (stems, leaves, and roots) of the transgenic tobacco line T3 in comparison with the non-transgenic control (CK) plants under NaCl stress.

With the stress level increase from 0 to 100 mmol L^−1^ NaCl, the control plant reduced root fresh weight 80.7% and root dry weight 66.9% (Table 1); In contrast, the transgenic T3 plants reduced by 34.3% and 29.6%, respectively. With the NaCl stress furthered to the 150 mmol L^−1^, the control plants did not produce root, while the T3 plants had fresh root weight 0.24 g and dry weight 0.03 g. Treatment effects on shoot fresh and dry weights tracked a similar tendency as the root traits (Table 1; Fig. 5). With the salt stress level added from 0 to 150 mmol L^−1^ NaCl, the non-transgenic plant reduced shoot fresh weight 62.1% and dry weight 56.7%, but the reduction were 15.2% and 19.6%, respectively, in the T3 line, significantly less than the control.

**Table 1:** Fresh and dry weights (g plant^−1^) of the transgenic plants (T3) with over-expression of *AtHKT1* gene and the non-transgenic control (CK) under various NaCl stresses. CK is the “CV87” non-transgenic tobacco cultivar.

NaCl concentration (mmol L^−1^)	Group	Root fresh weight	Root dry weight	Shoot fresh weight	Shoot dry weight
0	CK	0.96 ± 0.02 a	0.05 ± 0.00 b	3.51 ± 0.13 ab	0.33 ± 0.01 ab
	T3	1.00 ± 0.01 a	0.06 ± 0.00 a	3.62 ± 0.02 a	0.34 ± 0.00 a
50	CK	0.52 ± 0.01 c	0.03 ± 0.00 d	2.50 ± 0.04 c	0.28 ± 0.01 c
	T3	0.98 ± 0.01 a	0.05 ± 0.00 b	3.65 ± 0.05 a	0.33 ± 0.00 a
100	CK	0.19 ± 0.00 e	0.02 ± 0.00 e	1.92 ± 0.02 d	0.24 ± 0.00 d
	T3	0.65 ± 0.01 b	0.04 ± 0.00 c	3.40 ± 0.03 ab	0.31 ± 0.00 b
150	CK	0.00 ± 0.00 f	0.00 ± 0.00 f	1.33 ± 0.01 e	0.14 ± 0.00 fg
	T3	0.24 ± 0.01 d	0.03 ± 0.00 d	3.07 ± 0.04 b	0.27 ± 0.01 c
200	CK	0.00 ± 0.00 f	0.00 ± 0.00 f	1.34 ± 0.01 e	0.12 ± 0.00 fg
	T3	0.15 ± 0.01 e	0.02 ± 0.00 e	1.93 ± 0.30 d	0.21 ± 0.00 e
250	CK	0.00 ± 0.00 f	0.00 ± 0.00 f	1.32 ± 0.01 e	0.14 ± 0.00 fg
	T3	0.00 ± 0.00 f	0.00 ± 0.00 f	1.36 ± 0.00 e	0.14 ± 0.00 f
300	CK	0.00 ± 0.00 f	0.00 ± 0.00 f	1.15 ± 0.04 e	0.12 ± 0.00 g
	T3	0.00 ± 0.00 f	0.00 ± 0.00 f	1.08 ± 0.00 e	0.11 ± 0.00 g

T3 is the transgenic tobacco line with highest AtHKT1 gene over-expression. Tukey’s HSD test is used in analysis of significant differences (*P* < 0.05) in the NaCl-stress (n = 9, i.e., 3 runs × 3 replications).

### K^+^ and Na^+^ contents to salt stress

**Figure 6:**
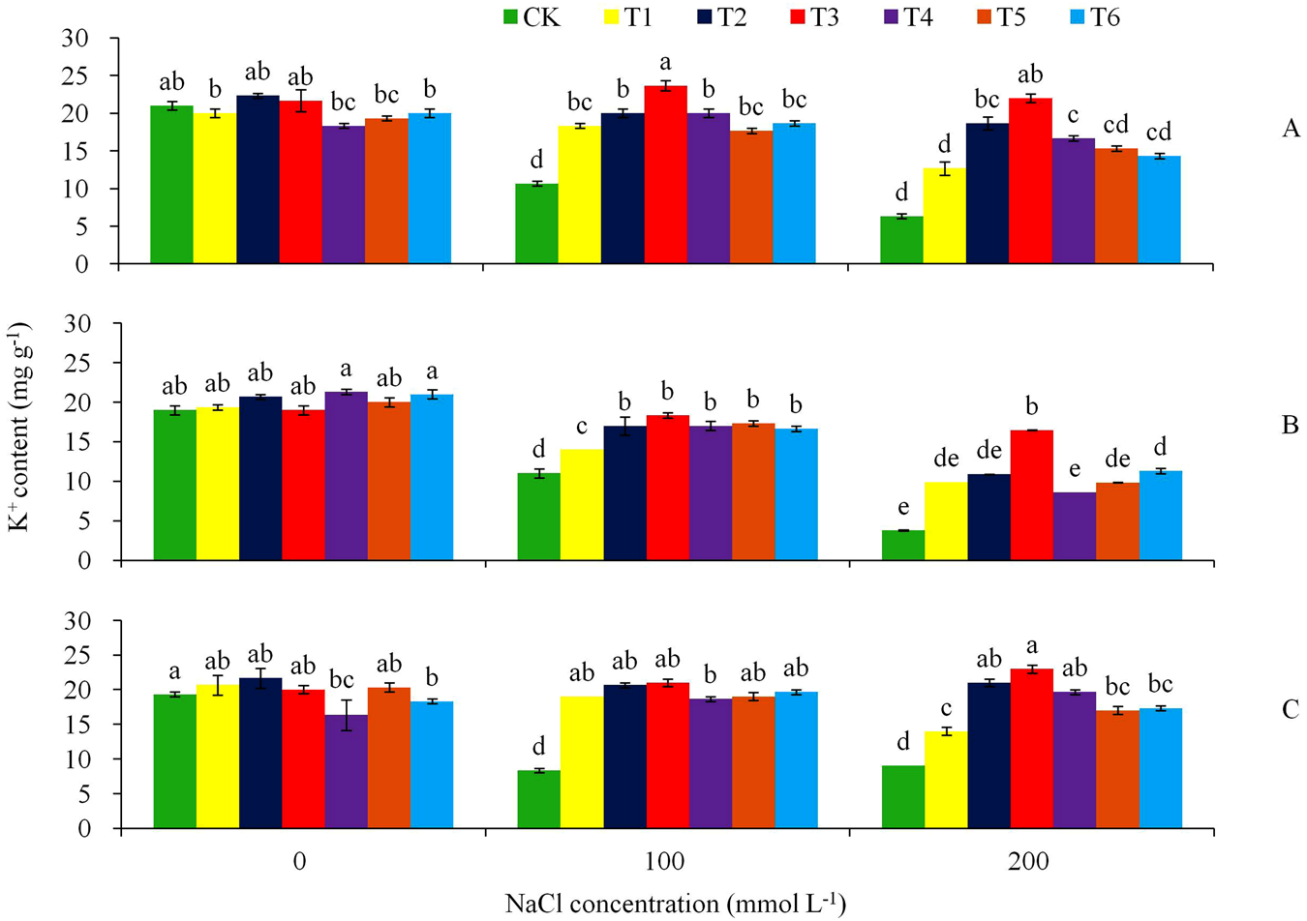
Effects of NaCl stress on (**A**) K^+^ in leaf, (**B**) K^+^ in stem, and (**C**) K^+^ in root for the CK and the transgenic lines. Tukey’s HSD test was used in analysis of significant differences (*P* < 0.05) in the NaCl-stress. The standard errors are shown in line bars (n = 9, i.e., 3 runs × 3 replications).

**Figure 7:**
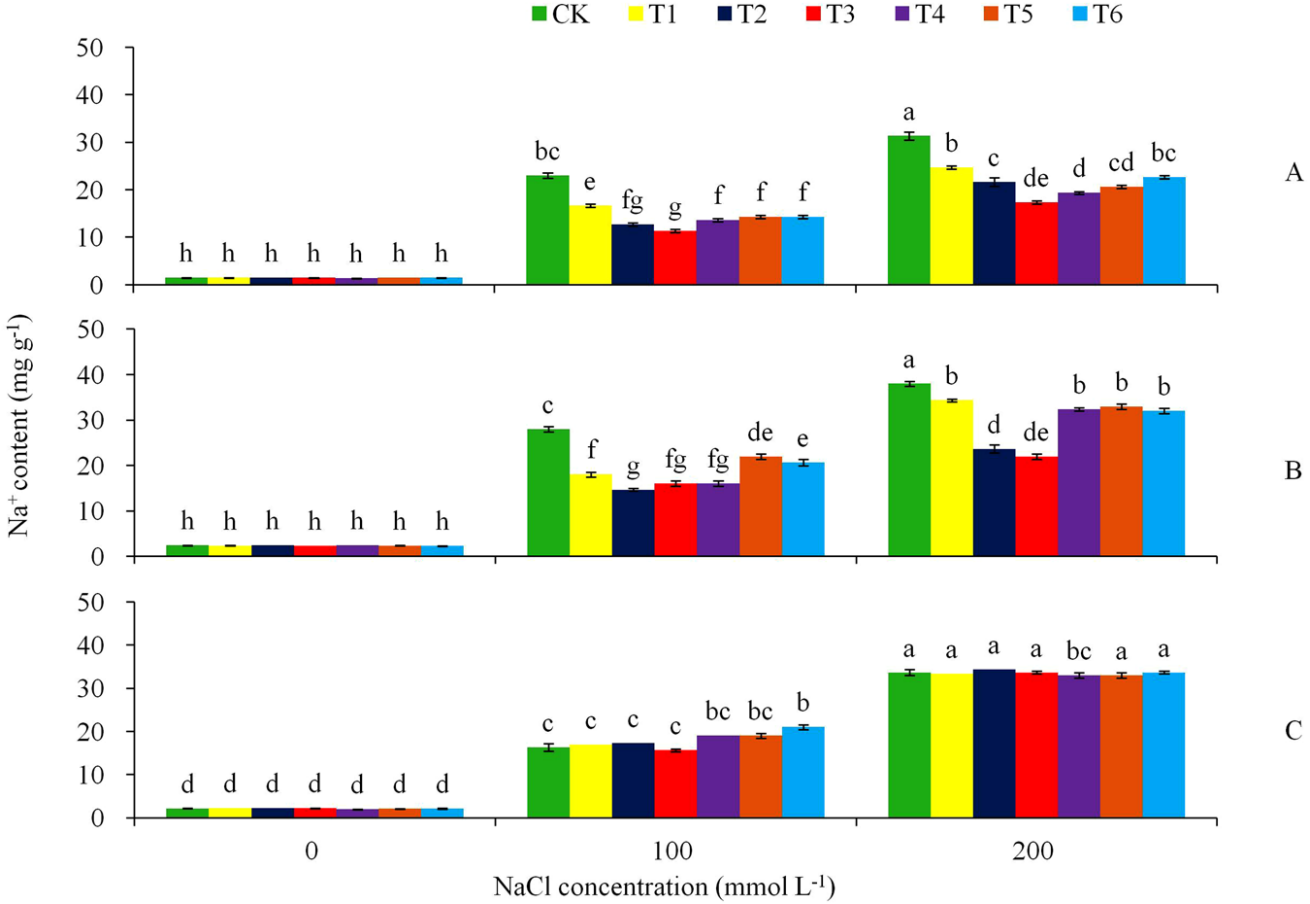
Effects of NaCl stress on (**A**) Na^+^ in leaf, (**B**) Na^+^ in stem, and (**C**) Na^+^ in root for the CK and the transgenic lines. Tukey’s HSD test was used in analysis of significant differences (*P* < 0.05) in the NaCl-stress. The standard errors are shown in line bars (n = 9, i.e., 3 runs × 3 replications).

To examine the effect of over-expression of *AtHKT1* gene in tobacco plant, we determined Na^+^ and K^+^ contents in roots, shoot and leaves under the NaCl stress and non-stress conditions (Figs 6 and 7). With the increase of NaCl stress, the control tobacco reduced K^+^ contents in the leaves, shoot and roots significantly (*P* < 0.05), while the values were stable in the transgenic T3 line (Fig. 6A–C). By Day 15 in the stress treatments, the leaves of the non-transgenic plants decreased K^+^ content from 21.0 mg g^−1^ of DW at the 0 mmol L^−1^ NaCl to 10.7 mg g^−1^ at the 100 mmol L^−1^ NaCl stress, furthered to 6.3 mg g^−1^ at the 200 mmol L^−1^ NaCl stress, representing a decrease of 49.1 and 70.0%, respectively (Fig. 6A). In contrast, the raise of the stress did not decrease K^+^ contents in the leaves of the transgenic lines T2, T3 and T5. In the stem and roots, the similar trend of treatment effect was found for K^+^ content (Fig. 6B–C): with the increase of the salt stress, K^+^ content in the control plants decreased significantly, while in the transgenic lines K^+^ content was nearly stable. Under the 200 mmol L^−1^ NaCl stress, K^+^ content in the leaves, shoot and roots decreased remarkably in the control plant, while the reductions were minimum in the transgenic lines. Furthermore, the salt stress treatments had significant effects on tissue Na^+^ (Fig. 7). With the increase of the salt stress from 0 to 200 mmol L^−1^ NaCl, the control plants increased Na^+^ contents more significantly (*P* < 0.05) than the transgenic plantlets in the leaves (Fig. 7A), stem (Fig. 7B), and roots (Fig. 7C). By Day 15 in the NaCl stress treatment, the control plants leaves raised Na^+^ content from 1.5 to 23.0 and furthered to 31.3 mg g^−1^ as the salt concentration increased from 0 to 100 and to 200 mmol L^−1^ NaCl (Fig. 7A). The average Na^+^ content in the control leaves was 1.4 to 2.0 times which of the transgenic plants at 100 mmol L^−1^, and 1.3 to 1.8 times at 200 mmol L^−1^ NaCl. Similarly, Na^+^ content in stem tacked a similar tendency of treatment influence as in the leaves (Fig. 7B). The salt treatments had a similar influence on the root Na^+^ contents in all the genotypes (Fig. 7C).

### Physiological and biochemical reactions in salt stress

**Figure 8:**
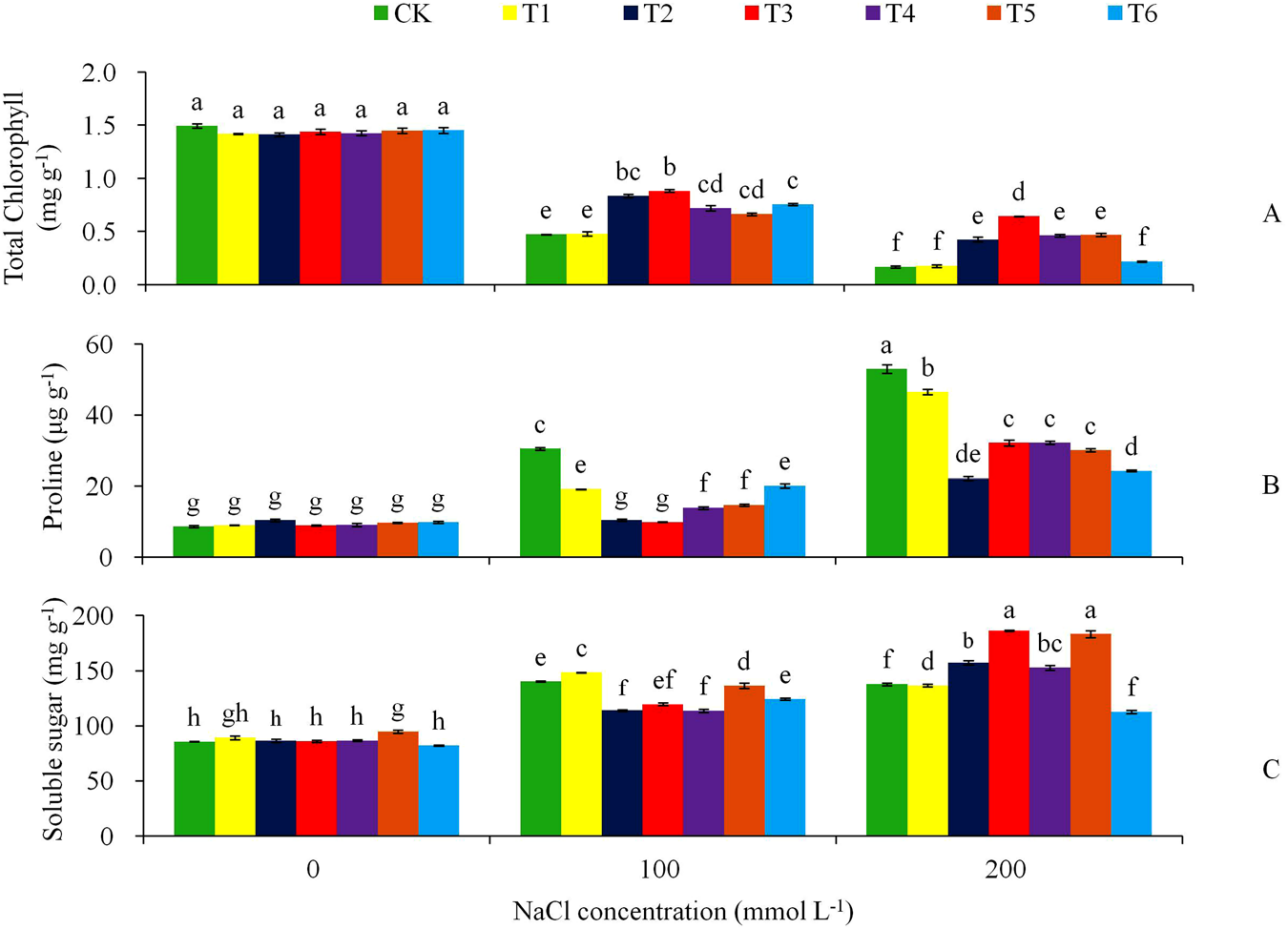
Effects of NaCl stress on (**A**) total chlorophyll content, (**B**) proline content, and (**C**) soluble sugar for the CK and the transgenic lines. Tukey’s HSD test was used in analysis of significant differences (*P* < 0.05) in the NaCl-stress. The standard errors are shown in line bars (n = 9, i.e., 3 runs × 3 replications).

The physiological and biochemical responses to salt stress were assessed by determining the contents of total chlorophyll, proline and soluble sugar, as well as malondialdehyde (MDA), electrolyte leakage, root activity, and the key enzymatic activities (peroxidase (POD), catalase (CAT) and superoxide dismutase (SOD)) in the leaves. With the raise of NaCl stress levels, total chlorophyll content reduced significantly (*P* < 0.05) in all the genotypes but the transgenic plants reduced significantly less than the non-transgenic plant (Fig. 8A). By Day 15 in the NaCl treatments, the non-transgenic plants decreased chlorophyll content from 1.5 mg g^−1^ at the 0 mmol L^−1^ NaCl to 0.5 mg g^−1^ at 100 mmol L^−1^, furthered to 0.2 mg g^−1^ at 200 mmol L^−1^ NaCl, representing a decrease of 66.7 and 88.6%, respectively, while the transgenic lines decreasing by 38.7–66.2% and 55.3–87.6%, respectively, the transgenic T3 line decreasing by 38.7 and 55.3,especially. On average, the transgenic plants total chlorophyll contents were 1.7 times that in the control at 100 mmol L^−1^ NaCl, and 2.4 times at 200 mmol L^−1^ NaCl. The proline content had a reverse trend as the chlorophyll: the non-transgenic plants raised proline content more obviously than the transgenic plants under NaCl stress (Fig. 8B). By Day 15 in the 100 mmol L^−1^ NaCl stress treatment, the proline content in the control plant was 30.5 μg**·**g^−1^ of FW, twice that of the transgenic lines; in the 200 mmol L^−1^ salt treatment, the control plant had proline content at 53.0 μg**·**g^−1^ FW, 45% more than those in the transgenic lines. Soluble sugar content were significant (*P* < 0.05) differences in the three levels of salt treatments, but at a given salt stress level the differences were inconsistent between the genotypes (Fig. 8C).

**Figure 9:**
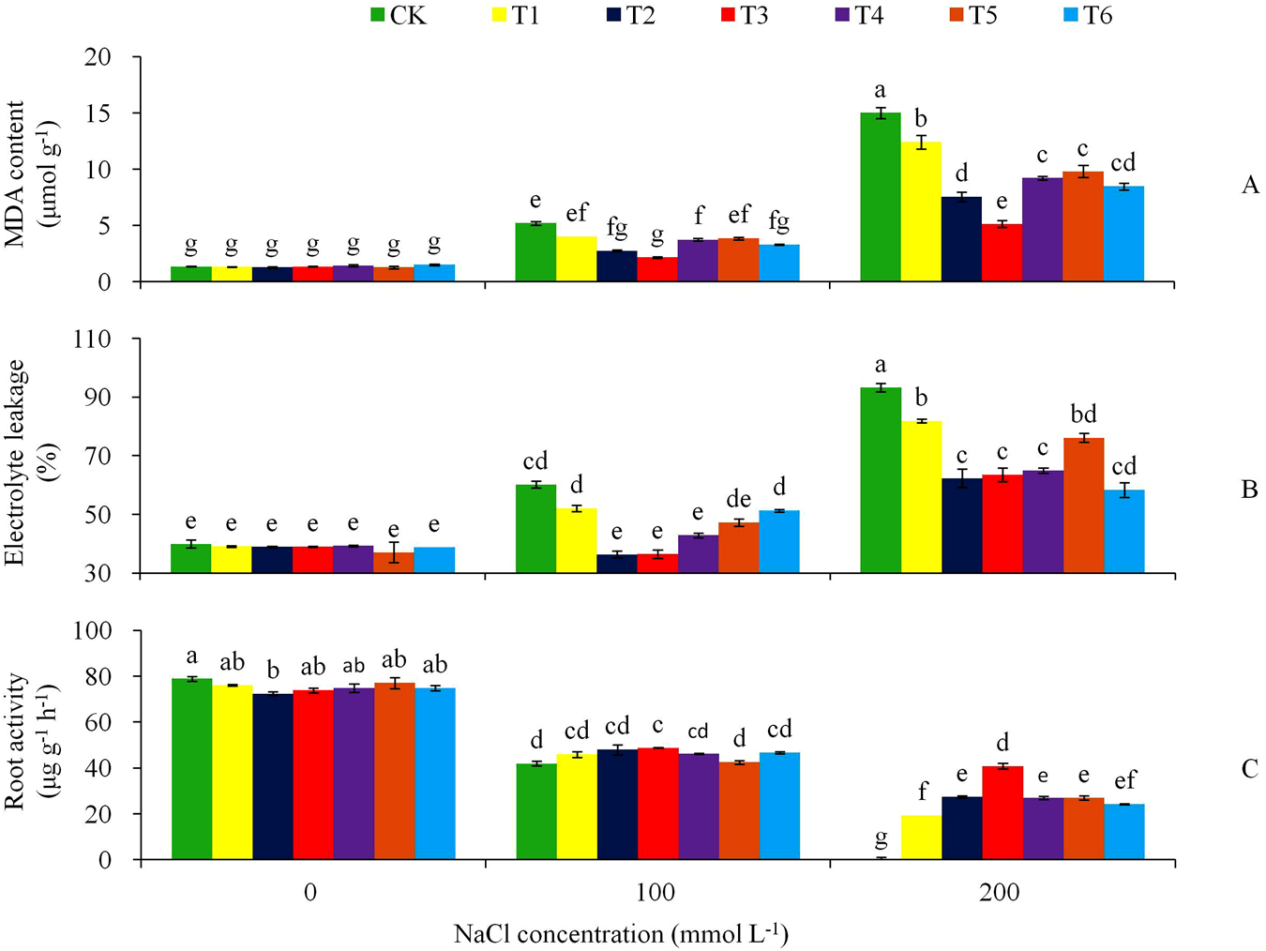
Effects of NaCl stress on (**A**) MDA, (**B**) electrolyte leakage, and (**C**) roots activity for the CK and the transgenic lines. Tukey’s HSD test was used in analysis of significant differences (*P* < 0.05) in the NaCl-stress. The standard errors are shown in line bars (n = 9, i.e., 3 runs × 3 replications).

The MDA content and electrolyte leakage followed a similar trend of treatment effects (Fig. 9A,B). By Day 15 under the NaCl stress, the non-transgenic plants raised MDA content from 1.3 μmol g^−1^ at the 0 mmol L^−1^ NaCl to 5.2 μmol g^−1^ at 100 mmol L^−1^ NaCl and furthered to 15.0 μmol g^−1^ at 200 mmol L^−1^ NaCl, representing a raise of 4.0 and 11.5 times, respectively (Fig. 9A). The transgenic lines had the lower amounts of MDA at the 100 and 200 mmol L^−1^ NaCl stress treatments. Similar to the MDA, electrolyte leakage in the non-transgenic plant raised more obviously with the increase of the NaCl concentrations than the transgenic plants (Fig. 9B). Opposite trend of treatment effects were shown in the root activity (Fig. 9C); the control plants reduced the root activity more significantly than the transgenic lines with the increase of the NaCl concentration. At the 200 mmol L^−1^ NaCl stress level, the roots activity of the control plants had a zero value, while the root activity improved effectively in the transgenic tobacco plants by *AtHKT1* gene over-expression.

**Figure 10:**
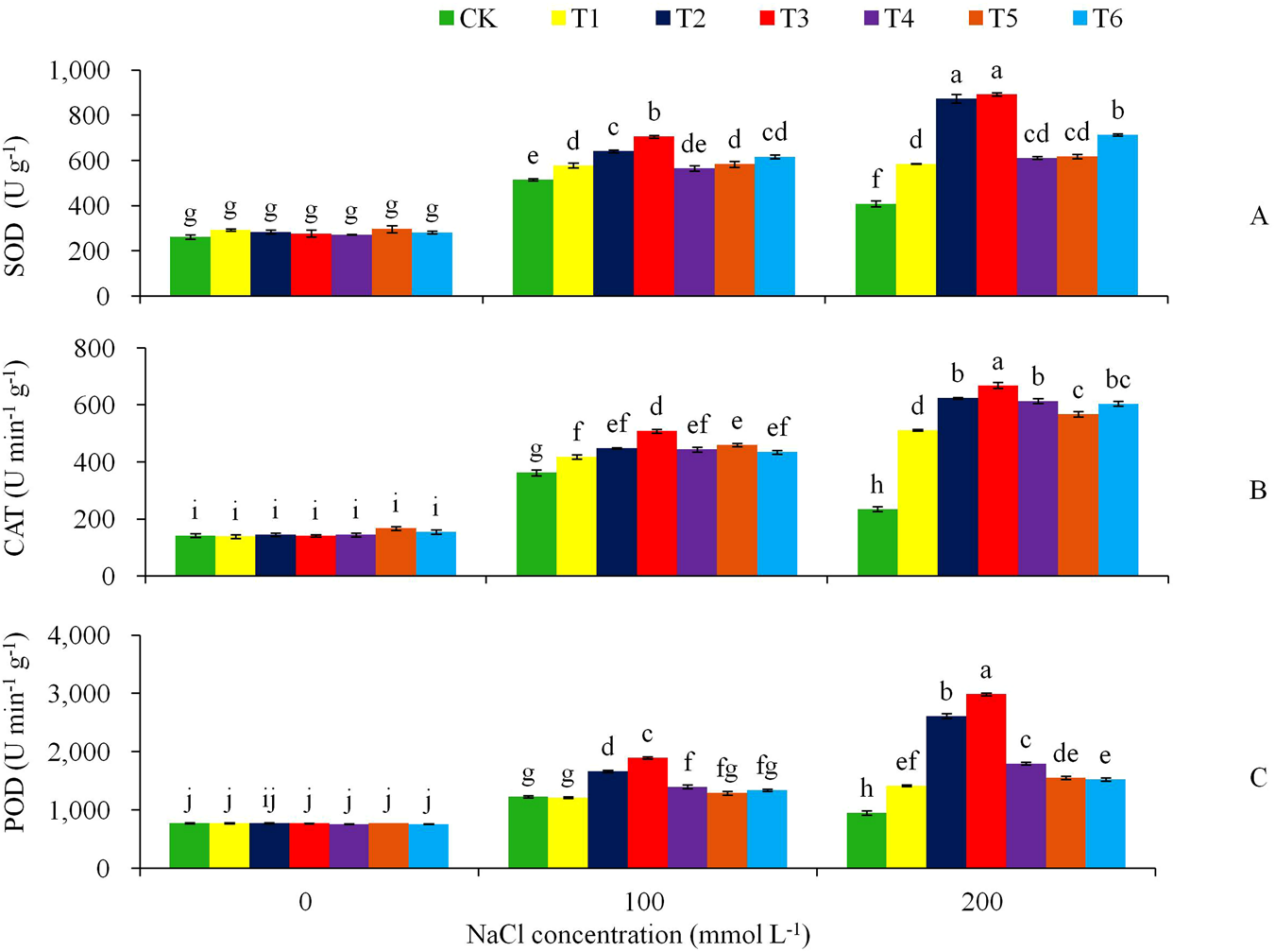
Effects of NaCl stress on (**A**) SOD, (**B**) CAT, and (**C**) POD for the CK and transgenic tobacco lines. Tukey’s HSD test was used in analysis of significant differences (*P* < 0.05) in the NaCl-stress. The standard errors are shown in line bars (n = 9, i.e., 3 runs × 3 replications).

Meanwhile, the biochemical detection revealed that the NaCl salt stress treatments stimulated the activity of the anti-oxidative enzymes SOD, POD and CAT (Fig. 10A–C). The highly significant differences between the transgenic and control plants were found under the 200 mmol L^−1^ NaCl stress for all these anti-oxidative enzymes, with the transgenic lines T2 and T3 having highest enzymatic activities among the genotypes.

## Discussion

High-affinity potassium transporters (HKTs) are known to play an significant effect in relieving salt stress in plants^31–34^. Our research team identified a high-affinity potassium transporter of *AtHKT1*gene and cloned it from the leaf of *Arabidopsis thaliana* in NaCl stress, and we found that *AtHKT1* gene sequence has an 84.0% similarity with *Thellungiella* HKT1 gene sequence (EF025500). The phylogenetic tree showed that AtHKT1 and *Thellungiella* HKT1 (ABK30935.1) were in the same cluster, suggesting that AtHKT1 may have the function of transporting K^+^ under salt stress. A number of studies have demonstrated that AtHKT1 controls Na^+^ accumulation in shoot^23–28^, but little has been reported if AtHKT1 might function in adjusting K^+^ content in heterologous plants. In this research, the *AtHKT1* gene was successfully transferred into a tobacco cultivar ‘CV87’. The *AtHKT1*gene was detected using several molecular methods, such as PCR, PCR-Southern blot, Southern blot, RT-PCR, and qRT-PCR. The *AtHKT1* has an open reading frame of 1521 bp and encods 506 amino acids. The gene was a stable alkaline protein with a possible theoretical isoelectric point of 9.33. One TrkH cation transporter protein conserved domain was at the 152–500 amino acids. The AtHKT1 also had ten transmembrane regions, five hydrophilic regions and thirteen hydrophobic regions with uniform distribution in the peptide. Our study demonstrated that *AtHKT1* was expressed in the root, stem and leaf vein xylem. Large differences in *AtHKT1* gene expression were discovered in the transgenic lines; the T3 line had the greatest *AtHKT1* expression, or 1.2 to 4.4 times than other five transgenic lines. These consequents mean that transformation of *AtHKT1* gene may give a chance for the enhancement of tobacco plants to salt resistance. The perspective is supported by the improved biophysicochemical features of the transgenic lines, including total chlorophyll, proline, soluble sugar, MDA, electrolyte leakage, root activity and antioxidant enzyme activities.

Recently, many research have been made to know the role of HKTs expression with the salt hypersensitivity in plant. *Zea mays* increased accumulation of Na^+^ in leaves with the natural *ZmHKT1* loss-of-function^35^. *Solanum lycopersicum* plants become hypersensitivity to salinity with *ScHKT1;2* and *SlHKT1;2* genes silencing^36^. In strawberry (*Fragaria ananassa*), an increase of *HKT1*expression in roots leads to high tolerance to salinity^37^. Most of them focused on the HKT1 function on Na^+^accumulation under salt stress. Our results add novel information that the over-expression of *AtHKT1* gene in tobacco leads to K^+^ steady state, reduces Na^+^ toxicity in leaf, improved physicochemical characters of the whole plant under salt stress; this may serve as a possible mechanism Potential impacts of the protein on the improvement of salt tolerance.

Damage to plants due to high salinity results in reduction of enzyme activity and loss of biomass due to osmotic imbalance caused by water deficit^6^. Keeping K^+^ in a high level under high Na^+^ stress conditions is identified as a strategy to enhance salinity tolerance^38^. Improving K^+^ content and decreasing Na^+^ accumulation could retrieve Na^+^ toxicity in leaves^39^, and this process may help alleviate osmotic imbalance and avoid salt-induced hydraulic failure via stomatal closure and a cascade of downstream effects^40^. In the present study, we measured K^+^ and Na^+^ contents in the different organs with or without *AtHKT1* expression under salt stress. We found that the K^+^ content was obviously more in the transgenic lines compared with the control plantlets under NaCl stress. The line with higher *AtHKT1* expression (the T3 line as an example) has stable K^+^ contents in the different organs under the different levels of NaCl stress. Under the 200 mmol L^−1^ NaCl stress, K^+^ content in the leaves, shoot and roots of the control plants decreased remarkably, while little reduction was shown in the transgenic lines. Furthermore, with increased salt stress, the control plantlets raised Na^+^ content significantly (*P* < 0.05) more compared with the transgenic lines in their leaves and stems. These results demonstrate that the transgenic plantlets with high *AtHKT1* expression are able to maintain K^+^ steady state under high Na^+^ stress conditions. This may suggest a possible mechanism that the enhanced tolerance to salt stress in the transgenic tobacco plants is due to the constitutive over-expression of *AtHKT1* gene helping keep K^+^ at a high level to minimize or avoid Na^+^ toxicity under salt stress. Also, the *AtHKT1* over-expression confers multiple functions, such as the improved activities of antioxidant enzymes SOD, CAT and POD; raised contents of chlorophyll and soluble sugar and root activity; and decreased proline and MDA contents and electrolyte leakage destruction. These enhanced physiochemical traits helped minimize cell damage under high salt concentrations, and consequently, increasing plant productivity.

This research examined the hypothesis that the over-expression of *AtHKT1* in tobacco maintains plantlet K^+^ accumulation under Na^+^ coercion, thus enhancing salt tolerance. Our results showed that under salinity stress, *AtHKT1* gene was expressed in the xylem of stem, root and leaf vein in the transgenic tobacco, which uninstalled Na^+^ from the shoot and leaves via transporting more K^+^, thus, decreased the toxicity of Na^+^ and advanced many biophysicochemical activities that assist decrease the salt-induced hurt. The constitution of *AtHKT1* in tobacco plants promoted a conversion from osmotic imbalance to osmotic balance. An over-expression of the *AtHKT1* improved the ability to keep plantlet growth even under high salt stress conditions. This study also provide reference for future research of crop salt tolerant improvement, over-expression *AtHKT1* gene in heterologous crop may be a useful method on increasing crop production.

## Methods

### Plant materials

The test was run at Gansu Agricultural University in 2012 and 2013. The plants of *Arabidopsis thaliana* plants were reproduced *in vitro* on 50 mL MS liquid medium. After culturing for 14 d, the ddH_2_O containing 160 mmol L^−1^ NaCl was added in the vitro. Plant leaves were collected after 12 h in the NaCl stress treatment for cloning of *AtHKT1* gene. The tobacco cultivar ‘CV87’ plants were reproduced *in vitro* by sub-culturing of single-node stem segments on MS medium. After being cultured for 20 d, the leaf slices of tobacco plants were sampled and used as the receptors of *Agrobacterium*-mediated transformation. Both *Arabidopsis thaliana* and tobacco plants were grown in a artificial climate chamber with 60–80 μmol m^−2^ s^−1^ light intensity and 8 h d^−1^ photoperiod at 22 ± 1 °C.

### Cloning of *AtHKT1* gene in *Arabidopsis thaliana*

Total RNA was isolated from *Arabidopsis thaliana* leaves with Trizol Reagent (Invitrogen, 15596026). Genomic DNA was digested by DNase I (TaKaRa, 2270 A). Five micrograms of total RNA were used for cDNA first-strand synthesis using the PrimeScript^TM^ 1st strand cDNA Synthesis Kit (TaKaRa, 6110A). All steps were according to the description of the manufacturer. The *AtHKT1* gene primers (Supplementary Table. S1) were designed based upon a published sequence of *Arabidopsis thaliana* HKT1 gene (GenBank accession No. AF237672). The cycling conditions (Supplementary Table. S1) were set up using a thermal cycler (UNO II, Biometra). Then the PCR products were cloned into the easy cloning vector pMD 18-T and reproduced in *Escherichia coli* DH5α for preservation and sequencing.

### Bioinformatics of *AtHKT1* gene

The AtHKT1 signal peptide and amino acid conservation were tested via InterProScan (http://www.ebi.ac.uk/Tools/InterProScan/). Gene similarity and phylogenetic tree analysis of AtHKT1 with other species HKTs were tested via MEGA5.0. AtHKT1 physical and chemical properties were analyzed using Protparam (http://expasy.org/tools/protparam.html). Transmembrane of AtHKT1 polypeptide was analyzed using TMHMM Serverv 2.0 (http://www.cbs.dtu.dk/services/TMHMM 2.0). Hydrophobicity of AtHKT1 polypeptide was analyzed using ProtScale (http://expasy.org/tools/protscale.html).

### Vector construction

The *β-glucuronidase* (*GUS*) gene was exchanged by 720 bp *GFP* gene in the plasmid pBI121, driven by CaMV 35S promoter, named pBI121-GFP. And then the 1521 bp cDNA of *AtHKT1* gene was inserted into the *Bam*H I-*Sma* I site of the plasmid pBI121-GFP named pAtHKT1 + GFP (Fig. 2A). The recombinant plasmid pAtHKT1 + GFP was transferred into *Agrobacterium tumefaciens LBA4404* by the method of freeze-thaw^41^. The plasmid was examined via PCR amplification and restriction enzyme digestion.

### Tobacco genetic transformation and molecular detection

Using *Agrobacterium*-mediated transformation, the leaves of the tobacco cultivar ‘CV87’ were used as the receptor^42^. The regenerated shoots of 1.5 cm in height were cut and cultured in the selective medium of inducing root (MS + 250 mg L^−1^ Carbenicillin +100 mg L^−1^ Kanamycin). Genomic DNA was extracted from leaves of control and transgenic plants by Edwards described^43^. The existence of *AtHKT1* gene was verified by normal PCR technique. The *AtHKT1* gene primers and the amplification program were shown in Supplementary Table. S1. For PCR-Southern, the PCR products in 0.8% agarose gel were denatured, and then shifted to a nylon membrane (Roche). The membrane was hybridized in hybridization buffer^44^. In Southern blot analysis, forty micrograms genomic DNA hydrolysis products with the endonuclease *Hin*d III were detached on 0.8% agarose gel. Hybridization methods were same as the method of PCR-Southern analysis described above. And then, cDNA were used in reverse transcription PCR assay (RT-PCR) analysis. Total RNAs was extracted and reverse transcription was performed using the PrimeScript^TM^ RT reagent Kit with gDNA Eraser (TaKaRa, RR047A) following the manufacturer’s instructions. The amplification methods, *AtHKT1* gene and *GFP* gene primers used in the expression determine were shown in Supplementary Table. S1.

### Gene expression analysis by Quantitative Real-Time PCR

Total RNA was isolated from non-transgenic (the control) tobacco and six transgenic tobacco line plants. The RNA quality inspection and quantified analysis was performed by a Nanodrop ND-1000 (Nanodrop, USA). Reverse transcription reaction was same as above. Quantitative Real-Time PCR amplification was performed using SYBR Premix Ex Taq^TM^ II (Tli RnaseH Plus) (TaKaRa, DRR820A) in 20 µL reaction mixtures on a Mx3005p QPCR system (Agilent, USA). Each qRT-PCR was repeated three replications. Water added in the reaction mixtures was as a blank control. The amplification methods, *AtHKT1* gene primers were shown in Supplementary Table. S1. After each reaction, the amplification specificity was confirmed by melt curve. The *AtHKT1* gene relative expression level was counted by 2^−ΔΔCt^.

### Expression of *AtHKT1* and *GFP* fusion genes in transgenic tobacco

The transgenic tobacco line T3 with high expression of *AtHKT1* gene in leaves, stem and roots was selected to make paraffin sections following the procedures described elsewhere^45^. Paraffin sections were verified using Leica DM6000B (Germany) fluorescence microscope automatic imaging system, and the excitation filter was I3 with blue light excitation.

### Physiochemical evaluations of *AtHKT1* gene transgenic tobacco

Transgenic tobacco lines taking the *AtHKT1* gene were assessed for their tolerance to NaCl stress treatments in tube. The transgenic tobacco line T3 with high expression of *AtHKT1* gene and the non-transgenic control were spread *in vitro* by sub-culturing of top single-node stem segments on MS medium added with different concentration NaCl. The top stem segments was cut from 20d old vitro plant and the length was 1.0 cm. The NaCl treatments were 0, 50, 100, 150, 200, 250, and 300 mmol L^−1^ NaCl concentrations. The test was run for three times. In each run, the treatments were set up in a entirely randomized arrangement with three replications. Every treatment in one replication had 3 bottles, one stem segment per bottle, with a total of 126 bottles (7 treatments × 2 lines × 3 replications × 3 bottles each). At Day 15 in the NaCl treatments, plant roots were separated from shoots and were weighed fresh weights separately. The samples dry weight were weighed after oven-dried to a consistent weight. The two of the NaCl concentrations (representing a high and low stress) were selectively used for further NaCl stress tests. Six transgenic tobacco lines carrying *AtHKT1* gene (T1, T2, … T6) and the non-transgenic control were used in the test. Sub-culturing of 1.0 cm top stem segment cuttings from 20d old vitro plant were cultured in MS liquid medium. At Day 15, plants with 3 leaves, 4.0–4.5 cm plant length, 3.0–3.5 cm root length, 0.5–0.6 cm stem thickness and same leave color were chosen for NaCl stress, the medium was replaced by 50 mL of MS medium with the different levels of NaCl. The plants were tested under three NaCl treatments: 0, 100, and 200 mmol L^−1^ NaCl. The examining was run for three times. In each run, the treatments were set up in a totally randomized arrangement in three replications. Every treatment of one replication had 10 bottles, one stem segment per bottle, with 630 bottles totally (3 treatments × 7 lines × 3 replications × 10 bottles each). The enough number of plants approved all tests to be carried out. At Day 15 after NaCl treatments, in each treatment, 30.0 g fresh leaves were sampled for the determinations of total chlorophyll content, proline content, soluble sugar contents, MDA, SOD, CAT and POD using the method described by Li^46^. Leaves, stem, and roots were oven-dried at 105 °C 10 min, and then at 65 °C to a constant weight and grinded separately. 0.3 g dry sample powders were selected for K^+^ and Na^+^ measure using the method described by Ghars^47^. 1.5 g fresh roots were taken for the determination of root activity using the triphenyl tetrazolium chloride method^44^ 6.0 g fresh leaves were sampled for the determinations of electrolyte leakage and RWC by the method of Yu^48^.

### Statistical analysis

The Tukey’s HSD test of SPSS Software 19.0 (SPSS, USA) was used in analyzing all data. Overall, there was a lack of treatment by run interactions for most of the variables evaluated, thus, the multiple runs data of the experiments were pooled in the statistical analysis. A probability of 5% was used to differentiate the significance between treatments or among the different categories of salt tolerance.

## Electronic supplementary material

Supplementary Information
