# Supplementary material for: AtHKT1 gene regulating K+ state in whole plant improves salt tolerance in transgenic tobacco plants
Source: Sci Rep. 2018 Nov 8;8:16585. doi: 10.1038/s41598-018-34660-9 (PMC6224463; doi:10.1038/s41598-018-34660-9)
Supplement: Supplementary file 1 — Supplementary Information [file 41598_2018_34660_MOESM1_ESM.pdf]

AtHKT1 gene regulating K^+ state in whole plant improves salt tolerance in transgenic tobacco plants

Li Wang[1, 2, *], Junlian Zhang[1, 3, *], Yuhui Liu[1,5], Shoujiang Feng[4], Zhuoyu Wang[6], Jinwen Zhang[5], Di Wang[1,5] & Yantai Gan[7]

[1]Gansu Provincial Key Laboratory of Aridland Crop Science, Gansu Key Laboratory of Crop Genetic and Germplasm Enhancement, Gansu Agricultural University, Lanzhou, 730070, China,

[2]College of Life Science and Technology, Gansu Agricultural University, Lanzhou, 730070, China,

[3]College of Horticulture, Gansu Agricultural University, Lanzhou, 730070, China,

[4]Institute of Soil, Fertilizer and Water-saving Agriculture, Gansu Academy of Agricultural Sciences, Lanzhou, 730070, China,

[5]College of Agronomy, Gansu Agricultural University, Lanzhou, 730070, China,

[6]Centre de Recherche CHUM, Montreal, H2X0A9, Canada, and

[7]Swift Current Research and Development Centre, Agriculture and Agri-Food Canada, Swift Current, S9H3X2, Canada

*Corresponding. wangl@gsau.edu.cn and zhangjunlian77@163.com

Keywords: *AtHKT1*, K^+ steady state, Osmotic balance, Salt tolerance, tobacco

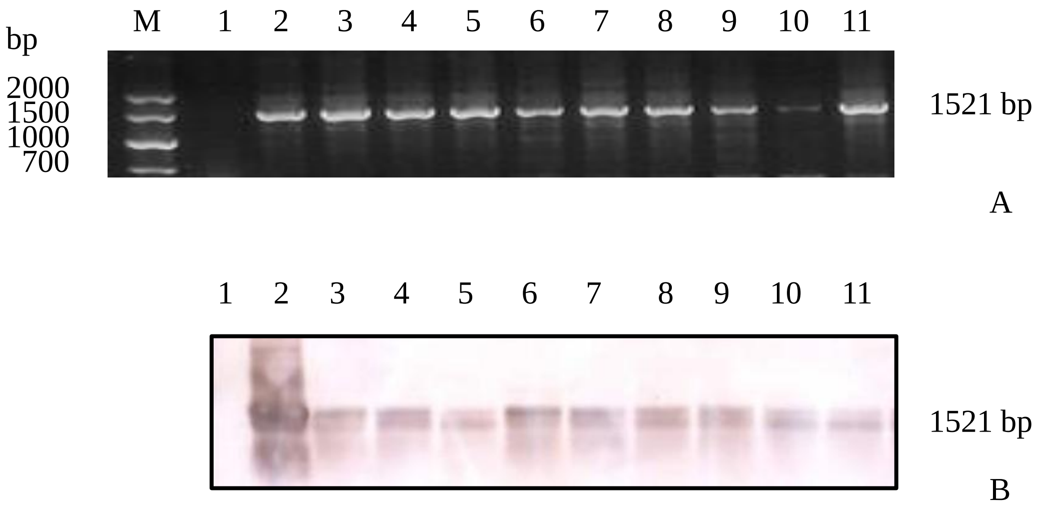

Figure S1. Verification of transgenic plants using (**A**) the PCR assay and (**B**) PCR-Southern of *AtHKT1* gene. M, DNA MarkerⅤ; 1, untransformed tomato plant as a negative control; 2, plasmid AtHKT1-GFP as a positive control; 3 to 11, transgenic tobacco lines. Hybridized with a *AtHKT1* probe labeled with digoxigenin. The grouping of gels/blots cropped from same part of the same gel.

Table S1. Genes primers, PCR and qPCR amplification program

Type	Genes	Primers	Amplification program	Notes
PCR and RT-PCR	*AtHKT1*	Forward primer: 5'-CGGGATCCATGGACAGAGTGGTGGCAAAAATAG-3' Reverse primer: 5'-CGCCCGGGTTAGGAAGACGAGGGGTAAAGTATCC-3'	1 min at 94 ℃; 35 cycles of 94 ℃ for 50 s, 53 ℃ for 50 s, 72 ℃ for 120 s, and 10 min final extension at 72 ℃.	Cloning of *AtHKT1* gene
	AtHKT1	Forward primer: 5'-CGGGATCCATGGACAGAGTGGTGGCAAAAATAG-3' Reverse primer: 5'-CGCCCGGGTTAGGAAGACGAGGGGTAAAGTATCC-3'	1 min at 94 ℃; 35 cycles of 94 ℃ for 50 s, 55 ℃ for 60 s, 72 ℃ for 90 s, and 10 min final extension at 72 ℃.	Transgenic plants PCR and RT-PCR detection
	GFP	Forward primer: 5'-ATGAGTAAAGGAGAACTTTTC-3' Reverse primer: 5'-TTATTATTTGTATAGTTCATCCATG-3'	3 min at 94℃; 35 cycles of 45 s at 94℃, 45 s at 59℃, and 60 s at 72℃, a final stage was for 5 min at 72℃.	Transgenic plants PCR and RT-PCR detection
qPCR	*AtHKT1*	Forward primer: 5' - ATC TGG CTC CTA ATC CCT CAA - 3' Reverse primer: 5' - CCG TCA CTC CAA GAA GAA CAC - 3'	30 s at 95℃; 40 cycles of 5 s at 95℃, and 34 s at 60℃	Gene expression assays with qPCR
	efla	Forward primer: 5'-CAA GGATGA CCC AGC CAA G -3' Reverse primer: 5'-TTCCTT ACC TGAACGCCT GT-3'	30 s at 95℃; 40 cycles of 5 s at 95℃, and 34 s at 60℃	Gene expression assays with qPCR, internal control gene
